# Supplementary material for: Cross-Cultural Adaptation and Validation of the Portuguese Version of the Psychosocial Impact of Dental Aesthetics Questionnaire
Source: Int J Environ Res Public Health. 2022 Aug 11;19(16):9931. doi: 10.3390/ijerph19169931 (PMC9408633; doi:10.3390/ijerph19169931)
Supplement: Supplementary file 1 [file ijerph-19-09931-s001.zip › ijerph-1823552-supplementary.pdf]

International Journal of
Environmental Research and Public Health

MDPI

Supplementary Materials

Table S1. Original and Portuguese versions of the PIDAQ questionnaire.

	Original	Portuguese
Item 1	I am proud of my teeth [R]	Tenho orgulho nos meus dentes [R]
Item 2	I like to show my teeth when I smile [R]	Gosto de mostrar os meus dentes quando sorrio [R]
Item 3	I am pleased when I see my teeth in the mirror [R]	Fico feliz quando vejo os meus dentes ao espelho [R]
Item 4	My teeth are attractive to others [R]	Os meus dentes são atrativos para os outros [R]
Item 5	I am satisfied with the appearance of my teeth [R]	Estou satisfeito(a) com a aparência dos meus dentes [R]
Item 6	I find my tooth position to be very nice [R]	Acho que a posição dos meus dentes é muito boa [R]
Item 7	I hold myself back when I smile so my teeth don't show so much	Eu contenho-me quando sorrio para que os meus dentes não apareçam tanto
Item 8	If I don't know people well I am sometimes concerned what they might think about my teeth	Se eu não conheço bem as pessoas, às vezes fico preocupado(a) com o que elas podem pensar sobre os meus dentes
Item 9	I'm afraid other people could make offensive remarks about my teeth	Temo que outras pessoas possam fazer comentários ofensivos sobre os meus dentes
Item 10	I am somewhat inhibited in social contacts because of my teeth	Sinto-me um pouco constrangido(a) nos contactos sociais por causa dos meus dentes
Item 11	I sometimes catch myself holding my hand in front of my mouth to hide my teeth	Às vezes, noto que coloco a mão à frente da boca para esconder os dentes
Item 12	Sometimes I think people are staring at my teeth	Às vezes acho que as pessoas estão a olhar para os meus dentes
Item 13	Remarks about my teeth irritate me even when they are meant jokingly	Comentários sobre os meus dentes irritam-me, mesmo quando são feitos em jeito de brincadeira
Item 14	I sometimes worry about what members of the opposite sex think about my teeth	Às vezes preocupo-me com o que pessoas que me atraem pensam sobre os meus dentes
Item 15	I envy the nice teeth of other people	Eu invejo os dentes bonitos de outras pessoas
Item 16	I am somewhat distressed when I see other people's teeth	Fico um pouco angustiado(a) quando vejo os dentes de outras pessoas
Item 17	Sometimes I am somewhat unhappy about the appearance of my teeth	Às vezes fico um pouco descontente com a aparência dos meus dentes
Item 18	I think most people I know have nicer teeth than I do	Acho que a maioria das pessoas que conheço tem dentes melhores do que eu
Item 19	I feel bad when I think about what my teeth look like	Eu sinto-me mal quando penso em como os meus dentes são
Item 20	I wish my teeth looked better	Eu gostava que os meus dentes parecessem melhores
Item 21	I don't like to see my teeth in the mirror	Não gosto de ver os meus dentes ao espelho
Item 22	I don't like to see my teeth in photographs	Não gosto de ver os meus dentes em fotografias
Item 23	I don't like to see my teeth when I look at a video of myself	Não gosto de ver os meus dentes quando assisto a um vídeo meu

[R] Indicates items that are reverse scored. These twelve items are rated on a response scale: 1 = "Not at all", 2 = "A little", 3 = "Somewhat", 4 = "Strongly", and 5 = "Very strongly" (in portuguese 1= "Não de todo"; 2 ="Um pouco"; 3 = "Às vezes"; 4 = "Concordo"; 5 = "Concordo totalmente").

Table S2. Test–retest reliability using ICCs for the PIDAQ-PT questionnaire.

	Cronbach's α Coefficient (95% CI)	ICC (95% CI)	p-Value
Item 1	0.99 (0.97; 1.00)	0.98 (0.96; 0.99)	<0.001
Item 2	0.97 (0.93; 0.99)	0.95 (0.91; 0.97)	<0.001
Item 3	0.94 (0.88; 0.97)	0.88 (0.80; 0.93)	<0.001
Item 4	0.93 (0.88; 0.97)	0.88 (0.79; 0.93)	<0.001
Item 5	0.91 (0.83; 0.95)	0.83 (0.72; 0.90)	<0.001
Item 6	0.90 (0.84; 0.95)	0.82 (0.71; 0.90)	<0.001
Item 7	0.77 (0.55; 0.91)	0.63 (0.43; 0.77)	<0.001
Item 8	0.78 (0.54; 0.95)	0.65 (0.46; 0.79)	<0.001
Item 9	0.87 (0.75; 0.94)	0.77 (0.63; 0.86)	<0.001
Item 10	0.91 (0.78; 0.98)	0.83 (0.72; 0.90)	<0.001
Item 11	0.85 (0.54; 0.97)	0.74 (0.59; 0.85)	<0.001
Item 12	0.79 (0.55; 0.91)	0.66 (0.47; 0.79)	<0.001
Item 13	0.86 (0.68; 0.94)	0.74 (0.58; 0.85)	<0.001
Item 14	0.80 (0.59; 0.91)	0.66 (0.47; 0.79)	<0.001
Item 15	0.82 (0.63; 0.93)	0.69 (0.52; 0.81)	<0.001
Item 16	0.76 (0.42; 0.92)	0.62 (0.41; 0.76)	<0.001
Item 17	0.87 (0.79; 0.93)	0.77 (0.63; 0.86)	<0.001
Item 18	0.79 (0.57; 0.90)	0.66 (0.46; 0.79)	<0.001
Item 19	0.87 (0.67; 0.96)	0.78 (0.64; 0.87)	<0.001
Item 20	0.90 (0.81; 0.96)	0.82 (0.70; 0.89)	<0.001
Item 21	0.90 (0.74; 0.98)	0.82 (0.71; 0.90)	<0.001
Item 22	0.84 (0.65; 0.94)	0.73 (0.57; 0.84)	<0.001
Item 23	0.92 (0.84; 0.97)	0.86 (0.77; 0.92)	<0.001

CI—confidence interval; ICC—intraclass correlation coefficient.

Table S3. Correlation between PIDAQ item scores.

Items	1	2	3	4	5	6	7	8	9	10	11	12	13	14	15	16	17	18	19	20	21	22	23
1	1.000	0.773 ***	0.825 ***	0.795 ***	0.804 ***	0.684 ***	-0.330 ***	-0.322 **	-0.317 **	-0.400 ***	-0.428 **	-0.209 *	-0.241 *	-0.207 *	-0.286 ***	-0.106 *	-0.538 ***	-0.426 ***	-0.517 ***	-0.563 ***	-0.543 ***	-0.523 ***	-0.503 ***
2	-	1.000	0.831 ***	0.771 ***	0.765 ***	0.642 ***	-0.467 ***	-0.411 **	-0.381 **	-0.461 ***	-0.454 **	-0.226 *	-0.298 *	-0.236 *	-0.332 ***	-0.160 *	-0.569 ***	-0.446 ***	-0.555 ***	-0.505 ***	-0.599 ***	-0.583 ***	-0.548 ***
3	-	-	1.000	0.846 ***	0.851 ***	0.696 ***	-0.399 ***	-0.348 **	-0.319 *	-0.429 ***	-0.419 **	-0.215 *	-0.269 *	-0.224 *	-0.337 ***	-0.138 *	-0.580 ***	-0.467 ***	-0.540 ***	-0.588 ***	-0.605 ***	-0.578 ***	-0.542 ***
4	-	-	-	1.000	0.812 ***	0.727 ***	-0.362 ***	-0.301 *	-0.268 *	-0.383 ***	-0.387 **	-0.130 *	-0.209 *	-0.171 *	-0.329 ***	-0.093 *	-0.547 ***	-0.435 ***	-0.496 ***	-0.595 ***	-0.546 ***	-0.531 ***	-0.498 ***
5	-	-	-	-	1.000	0.737 ***	-0.404 ***	-0.398 **	-0.313 **	-0.437 ***	-0.430 **	-0.218 *	-0.301 *	-0.253 *	-0.298 ***	-0.114 *	-0.604 ***	-0.435 ***	-0.585 ***	-0.616 ***	-0.599 ***	-0.565 ***	-0.535 ***
6	-	-	-	-	-	1.000	-0.323 ***	-0.284 **	-0.263 *	-0.369 ***	-0.331 **	-0.185 *	-0.180 *	-0.203 *	-0.277 ***	-0.084 *	-0.547 ***	-0.368 ***	-0.450 ***	-0.540 ***	-0.520 ***	-0.501 ***	-0.480 ***
7	-	-	-	-	-	-	1.000	0.696 ***	0.587 ***	0.653 ***	0.504 ***	0.365 ***	0.397 ***	0.406 ***	0.316 *	0.271 *	0.479 ***	0.482 ***	0.510 ***	0.392 ***	0.530 ***	0.529 ***	0.512 ***
8	-	-	-	-	-	-	-	1.000	0.723 ***	0.686 ***	0.570 ***	0.472 ***	0.516 ***	0.498 ***	0.307	0.294 **	0.495 **	0.474 **	0.547 ***	0.363 *	0.496 **	0.467 **	0.470 **
9	-	-	-	-	-	-	-	-	1.000	0.758 ***	0.633 ***	0.557 ***	0.594 ***	0.545 ***	0.270	0.330 **	0.488 **	0.464 *	0.598 ***	0.348 *	0.482 **	0.451 **	0.473 **
10	-	-	-	-	-	-	-	-	-	1.000	0.660 ***	0.539 ***	0.520 ***	0.496 ***	0.350 *	0.336 **	0.619 ***	0.537 ***	0.700 ***	0.427 **	0.614 ***	0.564 ***	0.559 ***
11	-	-	-	-	-	-	-	-	-	-	1.000	0.597 ***	0.604 ***	0.553 ***	0.333	0.332 **	0.552 ***	0.482 **	0.638 ***	0.397 *	0.510 **	0.530 **	0.508 **
12	-	-	-	-	-	-	-	-	-	-	-	1.000	0.494 ***	0.525 ***	0.244	0.292 **	0.375 **	0.355 *	0.490 ***	0.276	0.357 *	0.348 *	0.348 *
13	-	-	-	-	-	-	-	-	-	-	-	-	1.000	0.682 ***	0.248	0.364 **	0.493 **	0.408 *	0.581 ***	0.359 *	0.430 **	0.426 *	0.453 **
14	-	-	-	-	-	-	-	-	-	-	-	-	-	1.000	0.330	0.289 **	0.483 **	0.407 *	0.521 ***	0.328	0.378 *	0.393 *	0.416 *
15	-	-	-	-	-	-	-	-	-	-	-	-	-	-	1.000	0.387 **	0.468 ***	0.480 ***	0.393 ***	0.501 ***	0.415 ***	0.484 ***	0.427 ***
16	-	-	-	-	-	-	-	-	-	-	-	-	-	-	-	1.000	0.320 **	0.341 **	0.344 **	0.226 **	0.315 *	0.310 **	0.290 *
17	-	-	-	-	-	-	-	-	-	-	-	-	-	-	-	-	1.000	0.541 ***	0.716 ***	0.598 ***	0.693 ***	0.663 ***	0.644 ***
18	-	-	-	-	-	-	-	-	-	-	-	-	-	-	-	-	-	1.000	0.601 ***	0.579 ***	0.559 ***	0.596 ***	0.570 ***
19	-	-	-	-	-	-	-	-	-	-	-	-	-	-	-	-	-		1.000	0.558 ***	0.658 ***	0.613 ***	0.621 ***
20	-	-	-	-	-	-	-	-	-	-	-	-	-	-	-	-	-	-	-	1.000	0.557 ***	0.564 ***	0.551 ***
21	-	-	-	-	-	-	-	-	-	-	-	-	-	-	-	-	-	-	-	-	1.000	0.818 ***	0.772 ***
22	-	-	-	-	-	-	-	-	-	-	-	-	-	-	-	-	-	-	-	-	-	1.000	0.860 ***
23	-	-	-	-	-	-	-	-	-	-	-	-	-	-	-	-	-	-	-	-	-	-	1.000

* $p < 0.05$, ** $p < 0.01$, *** $p < 0.001$.
